# Multispectral Shared‐Aperture Metasurface With RF Wide‐Angle and Polarization Stability for Tri‐Band Transmission and High‐Fidelity Imaging

**DOI:** 10.1002/nap2.70171

**Published:** 2026-06-22

**Authors:** He Lin, Yingchi Lin, Ling Wang, Yuyao Wang, Ningrui Xiao, Da Mu, Xiaoliang Ma, Cheng Huang, Xiangang Luo

**Affiliations:** ^1^ State Key Laboratory of Optical Field Manipulation Science and Technology Institute of Optics and Electronics, Chinese Academy of Sciences Chengdu China; ^2^ School of Automation Engineering University of Electronic Science and Technology of China Chengdu China; ^3^ College of Materials Science and Opto–Electronic Technology University of Chinese Academy of Sciences Beijing China; ^4^ School of Optoelectronic Engineering Changchun University of Science and Technology Changchun China; ^5^ The 722 Research Institute of China State Shipbuilding Co. Ltd. Wuhan Hubei China

**Keywords:** low‐altitude aircraft, metasurfaces, multifunctional devices, optical transparency, radio frequency communication

## Abstract

The integration of multispectral, efficient, and stable radio‐frequency (RF) transmission and high‐fidelity imaging within a single optical window represents a critical yet challenging goal for low‐altitude aircraft (LAA) electro‐optical pods. Here, this paper proposes an optically transparent multispectral shared‐aperture metasurface (OMSM) with RF wide‐angle and polarization stability, which leverages a novel two‐dimensional spatially independent control strategy to overcome the aforementioned limitation. The unique mechanism unlocks superior dual‐mode optical and RF cross‐spectral performance. Design results demonstrate that it maintains high transmission efficiency across three bands (>−1 dB at 2–3 & 5–6 GHz and >−1.5 dB at 3.8–4.1 GHz) and stable in‐band interference suppression (average <−10 dB) within the 2–6 GHz spectrum. A minimal and independent set of low‐duty‐cycle RF modulating units works synergistically to deliver high transmittance across the visible (VIS) and near‐infrared (NIR) spectrum (> 94% at 380–1800 nm) with low imaging distortion. The prototype was fabricated using a large‐area low‐cost reproducible process, and the measured results show excellent agreement with the numerical simulations. This work provides a viable and easily implementable paradigm for designing cross‐spectrum cooperative devices.

## Graphical abstract

This study presents a multispectral compatible metasurface that achieves high transmission across three radio frequency bands, high optical transparency, and high fidelity imaging capabilities through a two‐dimensional spatial combination strategy of synergistic and minimalist low‐duty‐cycle units. It offers a minimalist and easily implementable approach for the design and application of multispectral cooperative devices in low‐altitude aircraft for the information age.

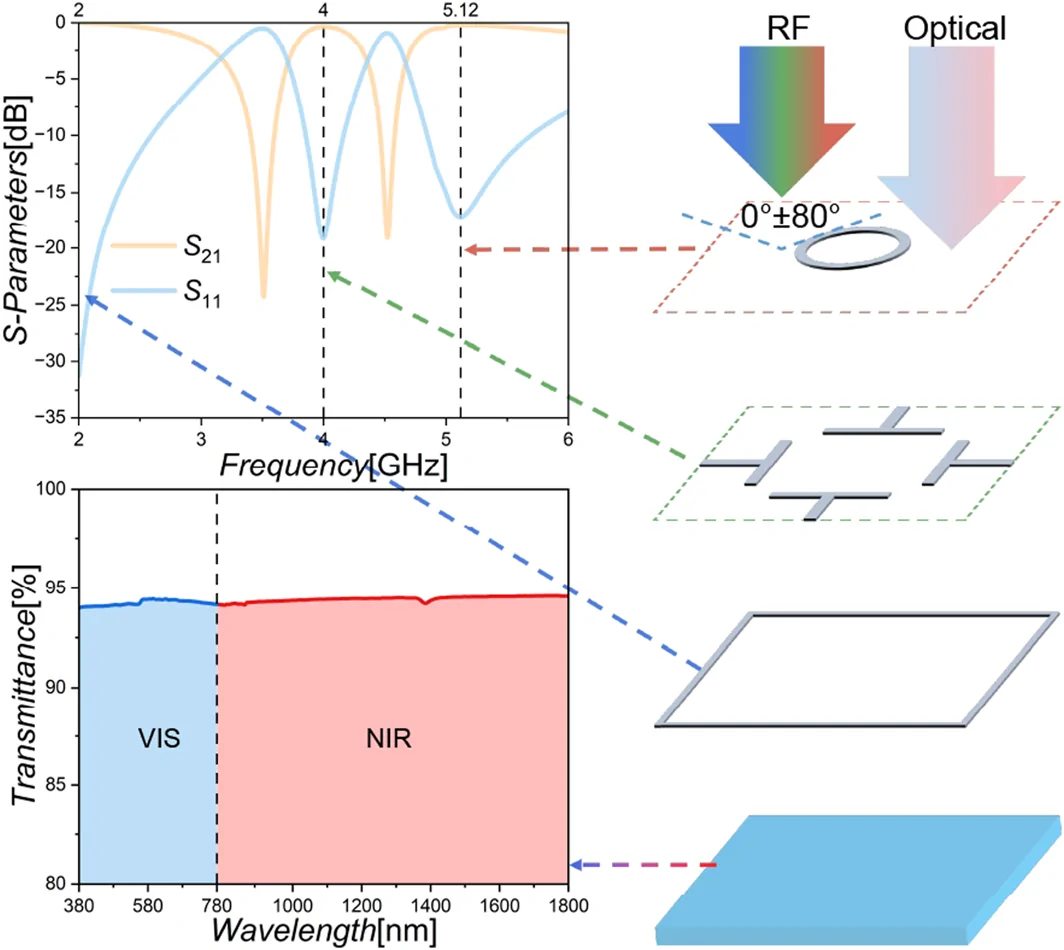

## 1 Introduction

Low‐altitude aircraft (LAA) have become indispensable for a wide range of applications in modern society, such as logistics [1], emergency response [2, 3], and aerial imaging [4]. However, the effectiveness and reliability of their aerial operations fundamentally hinge on stable and efficient onboard communication and sensing equipment. With both the rapid advancements of information technology and the widespread application of communication devices [5, 6, 7, 8], the LAA face formidable challenges to signal stability and anti‐interference capability, including malicious threats such as jamming [9, 10] and spoofing [11], as well as non‐malignant interference arising from spectrum congestion [12, 13, 14]. Conventional countermeasures, such as frequency‐hopping spread spectrum [15, 16, 17] and multiple‐input multiple‐output [18, 19, 20] technologies, can enhance link robustness. However, they often do so at the expense of increased system power consumption, complexity, and hardware burden, and they struggle to achieve cross‐spectrum cooperative management and functional integration at the physical layer. Particularly within the novel architecture of integrated design for aircraft optical windows (e.g., camera and sensor housings) and radio frequency (RF) windows (e.g., communication antennas), achieving functional compatibility and performance synergy across the RF and optical spectra has emerged as a pronounced technical bottleneck that demands urgent attention. Consequently, developing novel electromagnetic devices capable of integrating the functions of RF communication, interference blocking, and optical transmission at the physical level is of paramount importance for enhancing the mission efficacy of the next‐generation LAA in the information age.

In recent years, metasurface, the two‐dimensional planar structure composed of subwavelength artificial atoms arranged in specific configurations, has emerged as a canonical platform for unprecedented manipulation of electromagnetic waves [21]. The fundamental advantage of metasurfaces stems from their ability to tailor the amplitude [22], phase [23, 24, 25], polarization [26], and spectral response of electromagnetic waves at a deeply subwavelength scale via sophisticated meta‐atom design. This transformative capability underpins the evolution from static and passive to dynamically reconfigurable devices, which perform environment or command responsive beamforming [27, 28], modulation [29], and channel enhancement [30]. Consequently, they are regarded as a pivotal enabling technology for future 5 G/6G and integrated sensing and communication networks, providing a potent physical‐layer pathway toward unified communication and sensing. Despite the significant potential demonstrated by various types of metasurfaces [31, 32, 33], a more fundamental and critical challenge lies in achieving static multi‐functional and multi‐spectral compatibility when targeting practical platforms such as the LAA. Specifically, an ideal airborne multifunctional window must simultaneously satisfy the following stringent requirements: (i) High transmittance in the optical spectrum, such as the visible (VIS) and near‐infrared (NIR) bands, to ensure the proper operation of optical sensors for imaging and navigation; (ii) efficient signal transmission within specific RF communication bands (e.g., the common 2.4/5.8 GHz drone bands); and (iii) strong reflection or absorption in interference bands to suppress external interference. However, these physical demands are inherently contradictory: (i) Achieving broadband RF control typically relies on metallic resonant structures [34, 35, 36, 37], which severely obstruct optical transmission and (ii) conversely, the pursuit of high optical transparency often necessitates the use of transparent conductive materials such as indium tin oxide [38, 39, 40] whose limited electrical conductivity in turn constrains RF performance.

Ongoing research efforts are continuing to make progress in achieving multifunctional compatibility. For example, the design in ref. [41] achieved stable dual‐band transmission performance by attaching the metal metasurface structure onto a thin film. However, the thin film causes a slight decrease in optical transmission efficiency (VIS < 81%) and may affect transmission performance within other optical bands (e.g., the PET film exhibits absorption characteristics in the specific NIR bands). The approach of ref. [42] combined a honeycomb structure with multilayer metal‐dielectric films to achieve broadband optical transmission (VIS > 70%, NIR > 60%). However, it exhibits broadband RF transmission without multi‐band control capability. Significant challenges remain, as current solutions are hindered by either limited spectral functionality [43] or the need for complex multilayers [44, 45] and active control [46, 47, 48], preventing the simultaneous attainment of broadband RF filtering performance, ultra‐high optical transmission, structural simplicity, robustness, and wide‐angle stability.

To address these challenges, this work presents an optically transparent multispectral shared‐aperture metasurface (OMSM), designed to serve as a new type of integrated window for cross‐spectrum communication and optical detection for the LAA in the information age. The core innovation is a novel two‐dimensional spatial combination strategy of synergistic and minimalist low‐duty‐cycle units (SMLU). It should be emphasized that this design strategy is not a simple combination or superposition of independent resonators. Rather, its essence lies in employing a coplanar multi‐resonator structure with an extremely low metal duty cycle to achieve electromagnetic coupling synergy among the substructures—instead of mere functional superposition. This enables the simultaneous realization of stable RF selectivity and high optical transparency without requiring multilayer stacking or active components, thereby resolving the inherent trade‐off between these two properties. This distinction sets our work apart from the previous frequency selective surface and optically transparent metasurface, which typically rely on complex multilayers or compromise optical performance. The OMSM experimental sample, with dimensions of 355 × 355 ${\text{mm}}^{2}$ and a minimum structural linewidth of 0.05 mm at the very edge of the pattern, was fabricated using a low‐cost replicable large‐area wet etching process. Measurement results show the OMSM achieves efficient RF transmission (>−1 dB at 2–3 & 5–6 GHz, >−1.5 dB at 3.8–4.1 GHz) across a wide incident angle range, concurrent with stable interference rejection (average <−10 dB) and high broad‐spectrum optical transparency (> 94% at 380–1800 nm). Excellent imaging fidelity (with the difference of signal‐to‐noise ratio, SNR <0.3 dB and image contrast and IC < 1) is also demonstrated. Based on an innovative design concept, the OMSM achieves stable and efficient multiband‐compatible control performance through a simpler structure, fewer structural layers, and a low‐cost approach. This work shifts the paradigm from traditional single‐mode designs to a static multifunctional solution based on cross‐spectrum synergy, providing the LAA electro‐optical pods with a high‐performance and manufacturable option.

## 2 Design and Discussion

Figure 1 illustrates the conceptual diagram of the proposed SMLU‐based metasurface OMSM. The OMSM selectively transmits three specific frequency bands within the RF band—low (RF_low_), middle (RF_mid_), and high (RF_high_)—while maintaining exceptionally high transmittance across a broad optical spectrum (VIS and NIR). This design achieves synergistic integration of RF selectivity and optical transparency. This functionality is realized through the design of the SMLU. Each SMLU is essentially a co‐designed subwavelength resonant structure. Its geometric parameters (e.g., size, shape, and spatial arrangement) are meticulously optimized to excite desired resonant modes within the target RF bands (RF_low_, RF_mid_, and RF_high_), creating a stable transmission window. In contrast, within interference frequency bands, strong reflection—forming an effective interference suppression zone—is achieved via impedance mismatch or coupling effects among the units. Crucially, the conductive material constituting the units is patterned into microstructures that are significantly smaller than the communication wavelength, with an extremely low overall fill factor. This minimalist low‐duty‐cycle physical configuration ensures effective manipulation of RF while simultaneously minimizing blockage and scattering of incident optical waves, thereby preserving the inherent high optical transparency of the substrate material. In summary, the OMSM provides out‐of‐band suppression and can act as an intrinsic spatial filter, enabling the optimization of electromagnetic compatibility with the antenna architecture at the design stage. As a shared‐aperture window, it integrates RF functionality through physical‐layer co‐design; RF signals are spatially filtered and transmitted directly through it. The antenna can take the form of a low‐profile feed, sharing the same aperture with the optical system, thereby significantly reducing system integration complexity without altering the structure of the electro‐optical pod.

**FIGURE 1:**
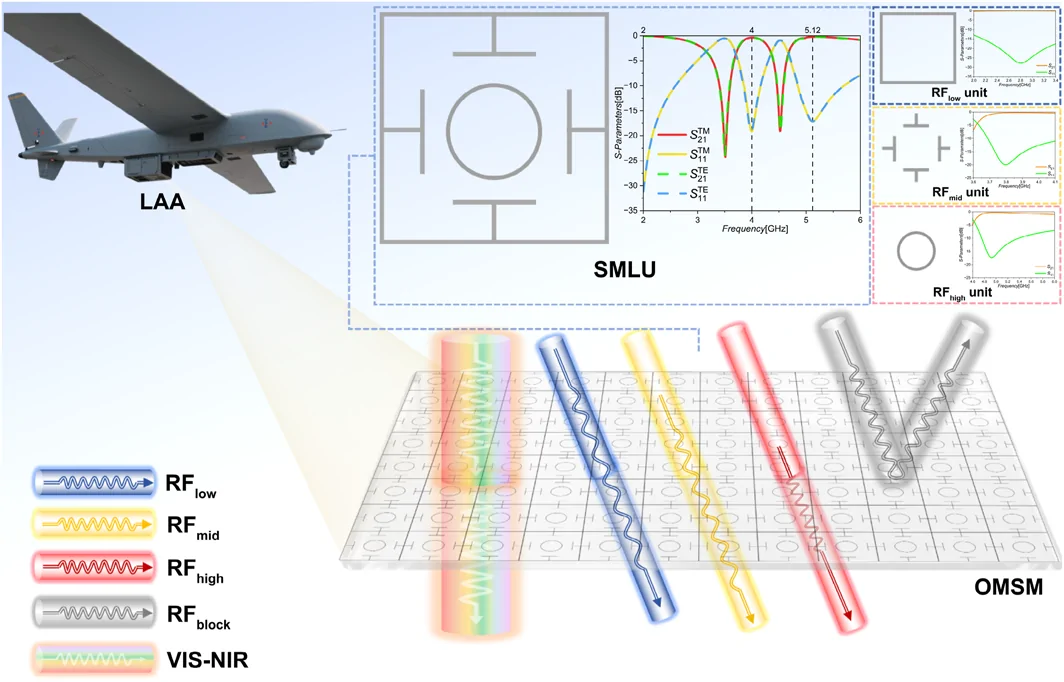
The application of the OMSM and its performance across the RF, VIS and NIR bands, including the SMLU model and primary substructures for different RF bands.

The structural model of the OMSM unit cell is shown in Figure 2a, which consists of a metal pattern and a glass substrate. The metal is aluminum, and the substrate is composite glass (ε=6.3(1−0.006i)). The structural parameters of the OMSM unit cell are: P=36 mm, w=0.1 mm, H=2 mm, $L_{1}=5$ mm, $L_{2}=10$ mm, and R=6.5 mm. The corresponding structure was analyzed using a full‐wave simulation. Figure 2b shows the transmission and reflection coefficients ($S_{21}$ and $S_{11}$) of the OMSM under normal incidence, showing three passbands and two blocking bands. In the low, middle, and high frequency bands, the bandwidths for which $S_{21}>-1$ dB are 2–3 GHz, 3.8–4.1 GHz, and 5–6 GHz, respectively. The tri‐band addresses the spectrum demands of diverse low‐altitude missions: the low band sustains secure remote control and high‐throughput intelligence backhaul, the middle band delivers high‐speed, low‐latency swarm coordination and mission data exchange, and the high band accommodates power‐sensitive short‐range high‐speed communication, collectively covering the core spectrum resources essential for low‐altitude operations. The transmission peaks of the bands are at $f_{1}=2$ GHz, $f_{2}=4$ GHz, and $f_{3}=5.12$ GHz.

**FIGURE 2:**
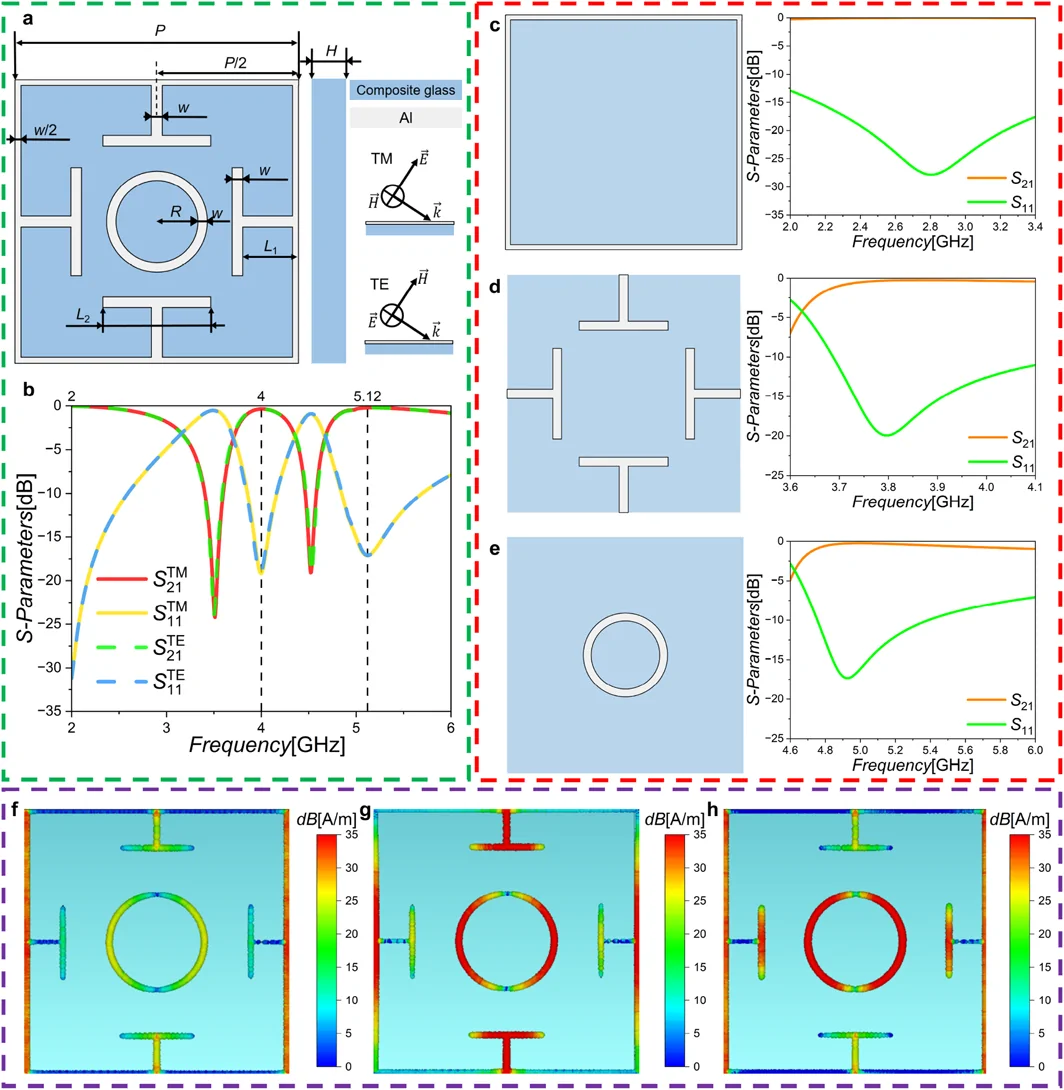
(a) Unit cell of the OMSM, including the SMLU parameters and composite glass thickness, with illustrations of TM‐ and TE‐polarized waves propagation. (b) The $S_{11}$ and $S_{21}$ of the OMSM under vertically incident 2–6 GHz. Primary substructure in the (c) low, (d) middle, and (e) high frequency bands, and corresponding S–Parameters. Surface current distribution of the OMSM unit under vertically incident TE‐polarized waves, corresponding frequencies of (f) $f_{1}=2$ GHz, (g) $f_{2}=4$ GHz, and (h) $f_{3}=5.12$ GHz.

To elucidate the transmission mechanism of the OMSM across three passbands, we examine the following three aspects: (i) the primary substructures at different bands; (ii) the surface current distribution at specific frequencies; and (iii) the influence of structural parameter variations on the $S_{21}$ and $S_{11}$. Because of the symmetric design of all substructures, their transmission is polarization‐independent. Consequently, the $S_{21}$ and $S_{11}$ plotted in Figure 2c–e are obtained by averaging the transverse electric (TE) and transverse magnetic (TM) polarized waves. The $S_{21}$ and $S_{11}$ for each substructure across other frequency bands are provided in Supporting Information S1: Note 1.

Substructure 1, the outermost square ring of the unit cell (Figure 2c), generates a wide high‐transmission band. Its $S_{21}$ exceeds −1 dB across 2–3.4 GHz, with a peak at $f_{1}^{\prime }=2.8$ GHz where the $S_{11}$ is below −30 dB. In contrast to the performance of the circular ring and T‐shaped structures (Supporting Information S1: Figure S1c,e), the square ring acts as the primary structure for the low frequency band. Substructure 2 (Figure 2d) is a T‐shaped structure, derived from a traditional cross and in contact with substructure 1 at its base. It exhibits $S_{21}$ above −1 dB from 3.8 to 4.1 GHz, peaking at $f_{2}^{\prime }=3.8$ GHz where $S_{11}$ is approximately −20 dB. The T‐shaped structure is confirmed as the primary resonator for the middle frequency band, as evidenced by its performance relative to the square and circular ring structures (Supporting Information S1: Figure S1a,f). Substructure three is a circular ring at the unit cell's geometric center (Figure 2e). Its $S_{21}$ remains above −1 dB within 4.8–5.8 GHz, with a peak at $f_{3}^{\prime }=4.9$ GHz and $S_{11}$ below −15 dB. The transmission performance of the square ring and T‐shaped structure in the high frequency band (Supporting Information S1: Figure S1b,d) indicates that the circular ring serves as the primary structure, ensuring high transmission in this band. The combination of these three substructures achieves the desired tri‐band transmission. This analysis of the primary substructures is consistent with the conclusions drawn from the subsequent surface current distribution analysis.

At $f_{1}=2$ GHz (Figure 2f), the surface current is primarily concentrated within the square ring structures matching the unit cell period. Here, the square rings function as the primary resonators, ensuring high transmission efficiency in the low frequency band. Although minor current concentration is also observed on the T‐shaped and circular ring structures, it does not affect the dominant role of the square rings as the main resonators. At $f_{2}=4$ GHz (Figure 2g), the surface current concentration occurs across all structures, indicating that the high transmission performance in the middle frequency band is collectively ensured by all substructures. Among these, the T‐shaped structure exhibits higher surface current density compared to other structures, confirming its role as the primary resonator at this frequency. At $f_{3}=5.12$ GHz (Figure 2h), similar to $f_{2}$, surface current concentration occurs across all structures, confirming that high transmission performance in the high frequency band is also collectively ensured by all substructures. Among these, the circular ring exhibits higher surface current density than other structures, indicating it serves as the primary resonator at this band. The above analysis indicates that the transmission of RF signals relies on the synergistic resonance of the entire structure, rather than a simple one‐to‐one correspondence between frequency bands and individual substructures.

Simulation results for different structural parameters under vertical incidence are shown in Figure 3. This process not only validates the analysis of surface current distribution and structural segmentation but also enables rapid parameter selection or design modification when adjusting the frequency bands. In addition, since the period P and linewidth w are global structural parameters, varying either of them changes the duty cycle and equivalent impedance of the unit cell, as well as the geometric characteristics of individual substructures, thereby affecting all three transmission bands (see Supporting Information S1: Note 2 for details). For such global structural parameters, they are given priority in this design: The unit cell period P is set to 36 mm, consistent with the dimensions of the square ring structure, while the linewidth w is ultimately set to 0.1 mm based on a balance between high optical transmittance of the OMSM and manufacturing cost. Therefore, parameter adjustment focuses on the circular ring and T‐shaped structures.

**FIGURE 3:**
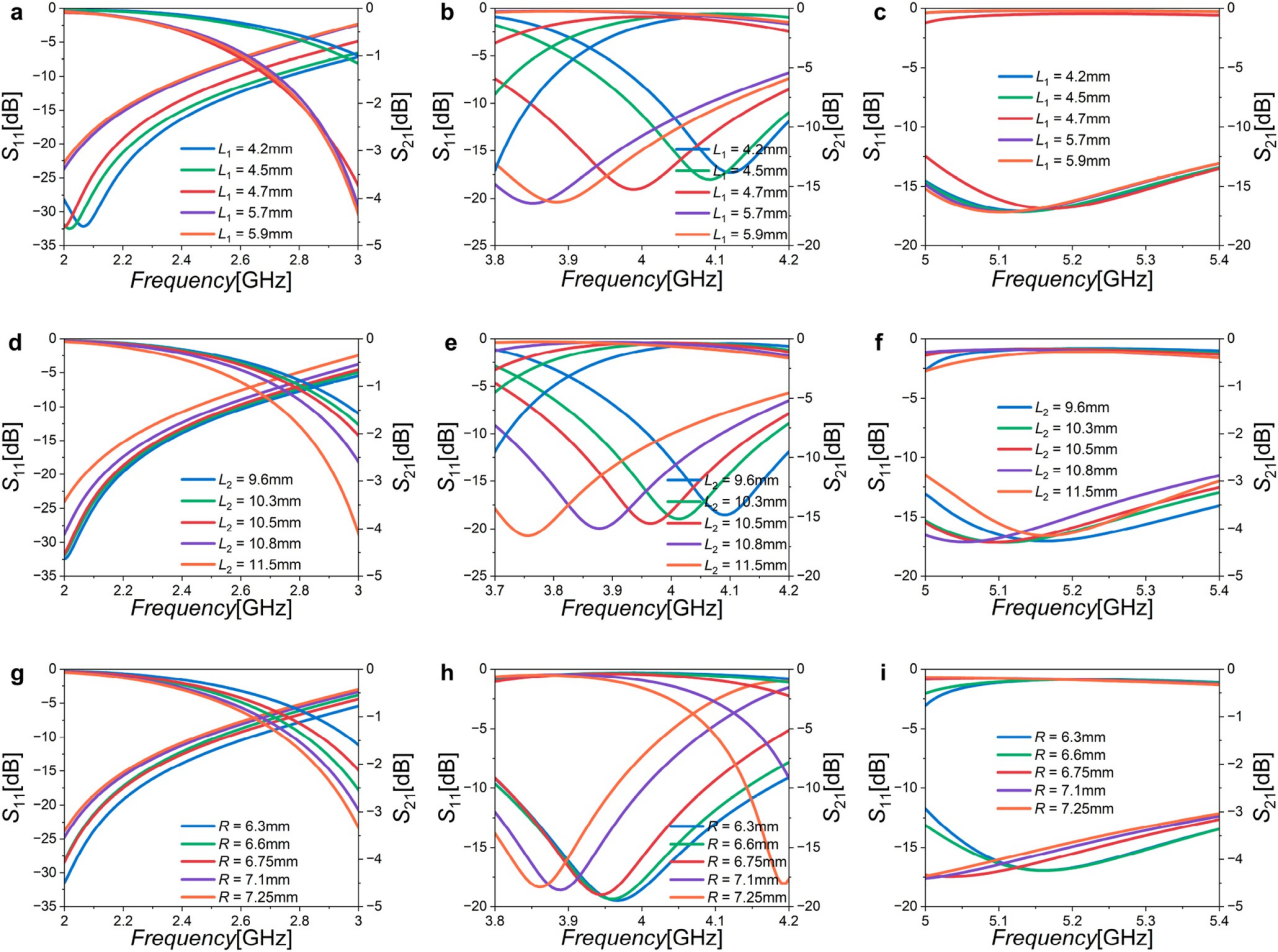
Effects of parameter variation on the value of $S_{11}$ and $S_{21}$ across different frequency bands: $L_{1}$ in the (a) low, (b) middle, and (c) high frequency bands; $L_{2}$ in the (d) low, (e) middle, and (f) high frequency bands; R in the (g) low, (h) middle, and (i) high frequency bands.

Figure 3a–i demonstrates control of transmission performance across three target frequency bands by adjusting the OMSM structural parameters. The data in Figure 3 represent the average over TE‐ and TM‐polarizations. Under normal incidence, it is challenging to directly evaluate the impact of parameter variations on transmission performance in the low and high frequency bands using the $S_{21}$ alone. To address this issue, we introduce the $S_{11}$ for a more intuitive assessment and extend its application to the middle frequency band to enable consistent and direct comparative analysis. Subsequent discussions on structural parameters will use the terms $L_{1}$, $L_{2}$, and R to denote their respective structures. As shown in Figure 2a, $L_{1}$ denotes the connector between the T‐shaped and square ring structures, $L_{2}$ represents the segment of the T‐shaped structure adjacent to the circular ring, and R signifies the circular ring structure.

As shown in Figure 3a–c, varying $L_{1}$ shifts the low frequency band by 0.19 GHz (at $S_{21}=-20$ dB) and tunes the peak of the middle frequency band across 3.85–4.12 GHz, whereas the high frequency band remains largely unaffected. This behavior aligns with the surface current distributions in Figure 2f–h. At the middle frequency band, $L_{1}$ participates strongly in the resonance, whereas at high frequencies, resonance is dominated by R and $L_{2}$, with minimal contribution from $L_{1}$. Consequently, $L_{1}$ primarily governs the performance of the low and middle frequency bands.

As shown in Figure 3d–f, variations in $L_{2}$ shift the low frequency band by 0.12 GHz (at $S_{21}=-20$ dB), tune the peak of the middle frequency band from 3.75 to 4.1 GHz, and shift the peak of the high frequency band between 5.05 and 5.18 GHz. Similar to $L_{1}$, this behavior is consistent with the surface current distribution shown in Figure 2f–h. $L_{2}$ exhibits stronger surface currents, and thus a more pronounced resonance, in the middle and high frequency bands compared to the low frequency band. Consequently, modifications to $L_{2}$ have the greatest impact on the middle frequency band, a significant effect on the high frequency band, and the weakest effect on the low frequency band.

Finally, as shown in Figure 3g–i, variations in R affect all three bands. The low frequency band shifts by 0.1 GHz (at $S_{21}=-20$ dB), whereas the peak frequencies of the middle and high frequency bands tune across 3.88–3.96 GHz and 5.0–5.18 GHz, respectively. This behavior is consistent with the surface current distribution in Figure 2f–h. R exhibits significant current density in the middle frequency band and the highest density in the high frequency band, reflecting its greater influence there. Consequently, the high frequency band exhibits the broadest tuning range, exceeding that observed for both $L_{1}$ and $L_{2}$.

In summary, varying $L_{1}$ primarily affects the low and middle frequency bands, with almost no influence on the high frequency band; varying $L_{2}$ significantly affects the middle and high frequency bands while having a weaker effect on the low frequency band; and varying R affects all three bands simultaneously. If the SMLU were merely an “incremental combination” of independent resonators, each parameter would affect only the frequency band corresponding to its respective substructure. However, the observed cross‐band coupling behavior precisely demonstrates the existence of electromagnetic synergy among the substructures—this is the clear demonstration of the synergistic design. This confirms that the SMLU framework is a synergistically coupled multi‐resonator system, rather than a simple incremental combination of three independent resonators. Furthermore, modifying the structural parameters essentially alters the current paths within the resonators, thereby enabling the tuning of resonant frequencies. This mechanism is confirmed by correlating the surface current distributions with the $S_{21}$ and $S_{11}$ under different parameter values. Moreover, multi‐parameter adjustments enable simultaneous shifting of all bands, as detailed in Supporting Information S1: Note 2.

To verify the superior angular performance of the designed OMSM, full‐wave simulations were also conducted. Figure 4 shows the $S_{21}$ for both TE‐ and TM‐polarized waves as a function of incident angle (0° to 80°) and frequency (2–3, 3.8–4.1, and 5–6 GHz), highlighting the performance advantage of the OMSM over the traditional window (TW, representing the plain composite glass) across angular and spectral domains. Figure 4g–l quantifies the RF transmission enhancement $\left(\Delta S_{21}\right)$ at $f_{1}$, $f_{2}$, and $f_{3}$, respectively, where $\Delta S_{21}=S_{21-OMSM}-S_{21-TW}$. These results highlight the superior angular performance of the OMSM. Furthermore, the OMSM produces slight phase differences only at the transmission band edges and under large incident angles, effectively avoiding signal distortion in wireless communication systems that could otherwise arise from polarization‐dependent phase disparities. See Supporting Information S1: Note 3 for details.

**FIGURE 4:**
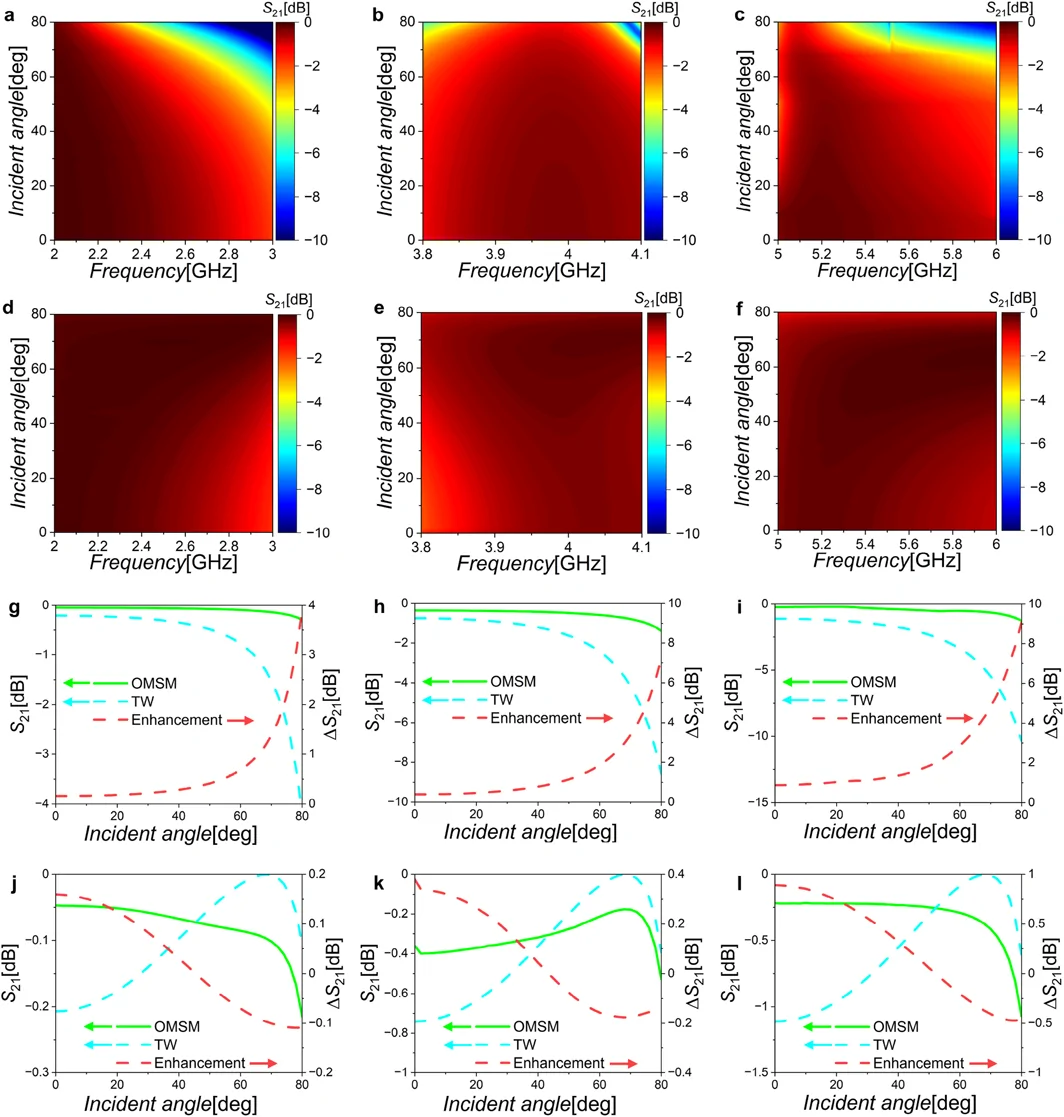
The relationship between the $S_{21}$ and incident angle, for TE‐ and TM‐polarized waves at (a, d) 2–3 GHz, (b, e) 3.8–4.1 GHz, (c, f) 5–6 GHz. Comparison of the performance of the TW and OMSM, for TE‐ and TM‐polarized waves at (g, j) 2 GHz, (h, k) 4 GHz, (i, l) 5.12 GHz.

As shown in Figure 4a,d, the OMSM exhibits exceptional angular stability in the low frequency band. Within the incidence angle range of 0° to 50°, TE‐polarized waves maintain high $S_{21-OMSM}$ across the entire band. When the incidence angle increases to 50°–80°, the $S_{21-OMSM}$ at the band edges gradually decreases; however, $S_{21-OMSM}$ remains above −1 dB near the $f_{1}$. In contrast, the $S_{21-OMSM}$ for TM‐polarized waves remains above −1 dB across all incidence angles and the entire frequency band. Figure 4g,j compare the transmission performance of the TW and OMSM at $f_{1}=2$ GHz across incident angles from 0° to 80°. For the TE‐polarized wave, the OMSM exhibits higher $S_{21}$ than the TW from 0° to 80° and the $\Delta S_{21}$ nearly reaches 4 dB at 80°. For TM‐polarized waves, the $S_{21-OMSM}$ is slightly inferior to $S_{21-TW}$ at incident angles beyond 45°, but it still remains above −0.3 dB.

As shown in Figure 4b,e, for the middle frequency band, both TE‐ and TM‐polarized waves maintain $S_{21-OMSM}$ above −1 dB at incident angles up to 60° within the entire frequency band, demonstrating robust angular stability. At larger incident angles (60°–80°), the behavior parallels that of the low frequency band: TM‐polarized waves retain high $S_{21-OMSM}$ across the entire band, whereas TE‐polarized waves remain high only near the $f_{2}$. Figure 4h,k compare the transmission performance of the TW and OMSM at $f_{2}$ = 4 GHz across incident angles from 0° to 80°. For TE‐polarized waves, the OMSM performs better than the TW across all incident angles, and the $\Delta S_{21}$ achieves nearly 7 dB at 80°. For the TM‐polarized wave, although the $S_{21-OMSM}$ is marginally lower than the $S_{21-TW}$ between 45° and 80°, it still maintains high efficiency with $S_{21-OMSM}$ consistently above −0.6 dB.

As shown in Figure 4c,f, the $S_{21}$ of both TE‐ and TM‐polarized waves remains above −1 dB across the high frequency band at incident angles up to 60°. However, within the 60°–80° range, their behavior diverges: TM‐polarized waves sustain high $S_{21}$ across the entire band, while TE‐polarized waves experience attenuation at the band edges, maintaining high $S_{21}$ only within a narrower band at $f_{3}$. Figure 4i,l compare the transmission performance of the TW and OMSM at $f_{3}=5.12$ GHz across incident angles from 0° to 80°. For TE‐polarized waves, the performance of the OMSM is enhanced across the entire range of incident angles compared to the TW, with the $\Delta S_{21}$ of nearly 9 dB at 80°. For TM‐polarized waves, high $S_{21-OMSM}$ is maintained above −1.1 dB; however, beyond 55°, the $S_{21-OMSM}$ for this polarization becomes slightly lower than $S_{21-TW}$.

Overall, at each peak frequency point, for TE‐polarized waves, the transmission performance of OMSM surpasses that of TW across the range from 0° to 80°, and the performance advantage gradually increases with the incident angle. The fundamental reason is that the designed metal pattern effectively compensates for the impedance mismatch that tends to deteriorate as the angle increases. For TM‐polarized waves, OMSM exhibits a slight advantage for incidence angles smaller than a frequency‐dependent threshold falling within 40°–50°, but becomes slightly inferior to TW for angles larger than that threshold. This is because near the Brewster angle, the TW itself exhibits extremely weak reflection and very high transmission efficiency, whereas the metal pattern of OMSM introduces a minor additional loss. OMSM effectively narrows the performance gap caused by polarization, while maintaining high efficiency for both polarizations, thereby providing stable and polarization‐insensitive high channel quality, which meets the core requirements of low‐altitude systems for link stability.

The preceding section explains the tri‐frequency operation mechanism of the OMSM and its capability for passband modulation. Subsequently, we present a theoretical analysis of the OMSM based on transmission line (TL) models.

The unit cell model of the OMSM in free space is shown in Figure 5a. To balance computational efficiency and accuracy, the specific geometric details of the metal pattern layer are neglected and replaced with an equivalent uniform surface impedance. This approach is also consistent with the fundamental assumption of TL theory, and the surface impedance is modeled by the TL circuit in Figure 5b. The admittance of this metal pattern layer, denoted as $Y_{m-p}$, is defined as $Y_{m-p}=G_{m-p}+jB_{m-p}$ and is illustrated in Figure 5c. To reduce computational complexity, $G_{m-p}$ is set to zero in calculations based on the low ohmic loss of metals. The impedance of the substrate is represented by $Z_{c-g}$. Using this equivalent circuit model, the electromagnetic response can be calculated with Equations ((1), (2), (3), (4), (5)) [41, 42, 43].

(1) $$M_{\mathrm{OMSM}}=M_{m-p}M_{c-g}=\left(\begin{array}{cc} A & B\\ C & D \end{array}\right)$$

(2) $$M_{m-p}=\left(\begin{array}{cc} 1 & 0\\ Y_{m-p} & 1 \end{array}\right)$$

(3) $$M_{c-g}=\left(\begin{array}{cc} \cos\delta_{c-g} & jZ_{c-g}^{TE/TM}\;\sin\delta_{c-g}\\ \frac{j}{Z_{c-g}^{TE/TM}}\sin\delta_{c-g} & \cos\delta_{c-g} \end{array}\right)$$

(4) δ=kHcosθ

(5) $$t^{\text{TE/TM}}=\frac{2\left(AD-BC\right)}{\left(A+\frac{B}{Z_{0}^{\text{TE/TM}}}+CZ_{0}^{\text{TE/TM}}+D\right)}$$

here k is the propagation constant, t is the transmission coefficient, H is the thickness of the substrate, $Z_{0}$ is the intrinsic impedance of free space (377 Ω), and θ is the propagation angle. For detailed Z of different polarization states and materials, refer to Supporting Information S1: Note 4.

**FIGURE 5:**
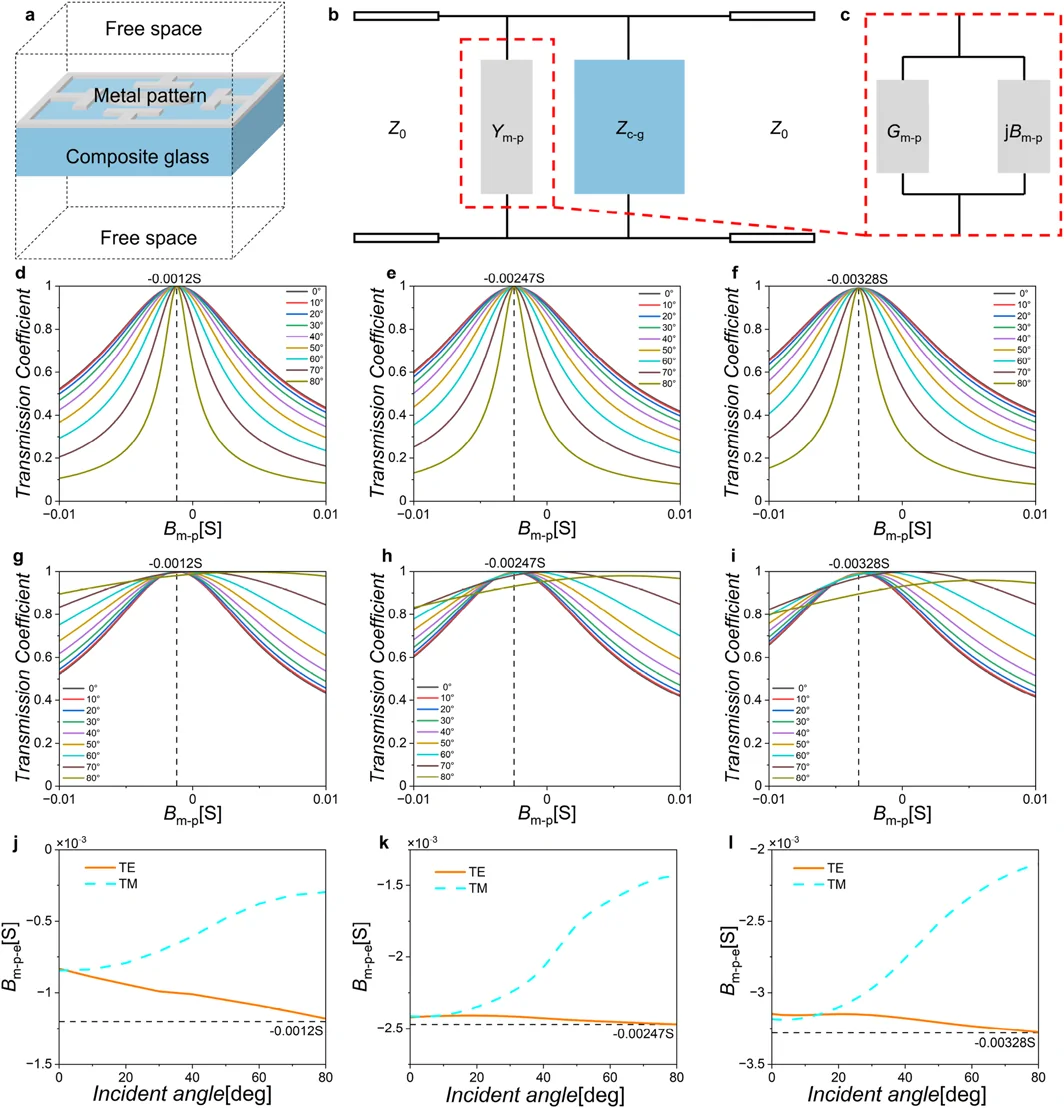
(a) The unit cell model of the OMSM in free space. (b) Corresponding TL model. (c) The impedance value $Y_{m-p}$ of the metal pattern can be divided into two parts, one part is the ohmic loss $G_{m-p}$, and the other part is the admittance $B_{m-p}$. The Relationship between $B_{m-p}$ and transmission efficiency for (d–f) TE‐ and (g–i) TM‐polarizations across 0°–80° incidence at $f_{1}=2$ GHz, $f_{2}=4$ GHz, and $f_{3}=5.12$ GHz. (j–l) $B_{m-p-e}$ versus incidence angle (0°–80°) from full‐wave simulations at $f_{1}=2GHz$, $f_{2}=4$ GHz, and $f_{3}=5.12$ GHz.

Based on TL theory, the optimal value of $B_{m-p}$ for achieving maximum transmission efficiency is derived, denoted as $B_{m-p-o}$. Figure 5d–i plot the transmission coefficient against $B_{m-p}$ for TE‐polarized and TM‐polarized waves at the peak frequencies of three bands ($f_{1}=2$ GHz, $f_{2}=4$ GHz, and $f_{3}=5.12$ GHz). For both TE‐ and TM‐polarized waves, the derived $B_{m-p-o}$ values are −0.0012 S at 2 GHz (Figure 5d,g), −0.00247 S at 4 GHz (Figure 5e,h), and −0.00328 S at 5.12 GHz (Figure 5f,i). The angular sensitivity to $B_{m-p}$ differs between the two polarization states, with TE‐polarized waves exhibiting the most pronounced variation. This phenomenon becomes increasingly pronounced as the incident angle increases.

Therefore, impedance matching for TE‐polarized waves is more challenging; high transmission efficiency of the OMSM is achieved only within a narrow range of $B_{m-p}$ values, particularly at incident angles greater than 60°. In contrast, TM‐polarized waves maintain high transmittance over a much broader range of $B_{m-p}$. To achieve high transmission efficiency for both TE‐ and TM‐polarized waves across a wide range of incident angles within the target frequency bands, we propose a metal pattern that provides a $B_{m-p}$ that approximating $B_{m-p-o}$ at the target frequency.

The equivalent circuit model indicates that a ring‐shaped structure is required to yield the desired negative $B_{m-p}$ value [49]. Specifically, under incident electromagnetic wave excitation, a complete ring structure (such as a square or circular ring) generates induced currents that form a closed loop, producing an inductive response and thus a negative surface admittance value. By contrast, a pure patch structure exhibits capacitive susceptance, with the corresponding surface admittance value being positive. To achieve a multi‐resonance response, we incorporated multiple resonators, as shown in Figure 2c–e. The T‐shaped structure is connected to the square ring, providing an additional current path while maintaining its essential inductive ring‐like behavior. For details on the method of extracting the $B_{m-p}$, refer to Supporting Information S1: Note 4.

The admittance value of the metal pattern, extracted from full‐wave simulation data, is denoted as $B_{m-p-e}$. Owing to the rigorous adherence to the mathematical relations of the TL model throughout the extraction procedure, reinserting the $B_{m-p-e}$ into the model yields a computed transmission spectrum that is expected to exhibit exceptional concordance with the full‐wave simulated counterpart near the three designated passbands. Furthermore, the mutual coupling effects among the substructures are not neglected—this can be corroborated by the surface current distribution shown in Figure 2. Moreover, since the TL model treats the entire patterned metal layer as an equivalent impedance, the impedance contribution from mutual coupling is already incorporated in this value. Figure 5j–l show the curves of the metal pattern's $B_{m-p-e}$ versus incident angle at $f_{1}$, $f_{2}$, and $f_{3}$, respectively. At vertical incidence, slight deviations exist between the $B_{m-p-e}$ and $B_{m-p-o}$ for both TE‐ and TM‐polarized waves. As shown in Figure 5d–i, these deviations have negligible effects on transmission efficiency. As the incident angle gradually increases, the $B_{m-p-e}$ of the TE‐polarized wave progressively approaches $B_{m-p-o}$. This behavior aligns with the trend where the $B_{m-p}$ range corresponding to high transmission efficiency for TE‐polarized waves narrows with increasing angle, thereby helping maintain high transmission efficiency for TE‐polarized waves at high incident angles. For TM‐polarized waves, $B_{m-p-e}$ progressively deviates from $B_{m-p-o}$ as the angle increases. As shown in Figure 5g–i, the curves of the TM‐polarized wave's $B_{m-p}$ as a function of the transmission coefficient at different incident angles indicate that the $B_{m-p}$ corresponding to peak transmittance gradually deviates from the initial $B_{m-p-o}$ as the angle increases. This deviation is beneficial for maintaining high transmission efficiency of the TM‐polarized wave over a wide range of incident angles. In summary, this design achieves high transmission efficiency across both polarization states and a wide range of incident angles, which meets the design objectives.

## 3 Preparation and Experiment

To systematically characterize the RF and optical compatible regulation performance of the OMSM, an experimental sample with overall dimensions of 355× 355 ${\text{mm}}^{2}$ was fabricated. The sample was built on a 2 mm thick glass substrate, with the metallic pattern formed via a large‐area wet etching process. More importantly, the process exhibits superb structural fidelity, accurately transferring the designed SMLU geometric patterns (including the critical low‐duty‐cycle and elongated structures) to the metal thin film, with a processing tolerance controlled within 0.01 mm (i.e., ±0.005 mm of the design value). This processing tolerance only slightly affects the RF performance of the designed sample, as detailed in Supporting Information S1: Note 5. The fabrication process that enables this performance is outlined in Figure 6a. The process begins with the deposition of a uniform aluminum film on a cleaned glass substrate. Compared to other metallic materials, aluminum offers superior oxidation resistance, along with the advantages of low cost, ease of processing, and high precision. This is followed by standard photolithography steps: photoresist coating, exposure, and development, to accurately transfer the SMLU pattern. The critical wet etching step then removes the unprotected metal with high fidelity, achieving the designed subwavelength features (with a minimum feature linewidth of 0.05 mm, i.e., at the very edge of the pattern) across the entire 355 × 355 ${\text{mm}}^{2}$ area. Subsequent photoresist stripping and a final cleaning step yield the stable OMSM device. Employing this high‐fidelity fabrication process maximally preserves the structural and functional integrity of the device, thereby ensuring consistent performance in real‐world application scenarios. This is fully validated by the tolerance analysis and durability tests presented in Supporting Information S1: Note 5. As shown in Figure 6b, the OMSM sample exhibits high optical transparency and maintains excellent visual transmission performance under working conditions, providing intuitive evidence that the metasurface pattern has a negligible impact on imaging quality. The magnified microscopic inset reveals the slender low‐duty‐cycle geometry of an individual SMLU, which forms the foundation for its multispectral functionality.

**FIGURE 6:**
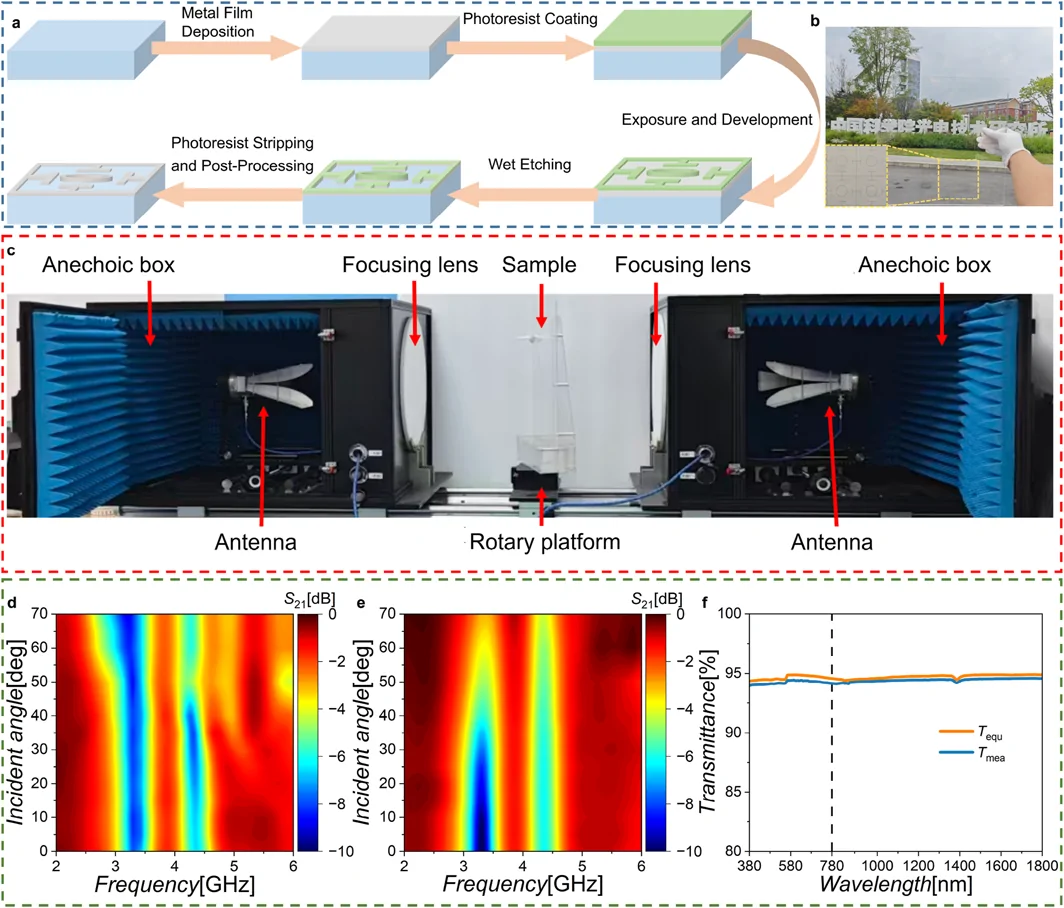
(a) Fabrication process flow of the OMSM. (b) View through the OMSM and its unit cell structure. (c) Experimental setup for RF transmission efficiency measurement using the free‐space method. Measured the $S_{21}$ as a function of frequency and incident angle for (d) TE‐ and (e) TM‐polarized waves. (f) Measured and theoretically equivalent optical transmittance data.

The measurement setup is illustrated in Figure 6c. First, a linearly polarized antenna is placed within an anechoic box lined with microwave‐absorbing material to minimize measurement errors resulting from RF leakage. Then, two such boxes are positioned symmetrically on opposite sides of the OMSM sample. Focusing lenses are installed at the apertures of the anechoic boxes. The transmitter‐side lens focuses the incident beam onto the sample, while the receiver‐side lens focuses the transmitted beam onto the receiving antenna to minimize loss, thereby improving measurement accuracy. The OMSM sample is mounted on an acrylic stand, which is in turn attached to a motorized rotation stage with a minimum step angle of 0.01°. The rotation stage is program‐controlled to precisely adjust the angle between the sample's normal and the incident beam, enabling continuous angular scanning and measurement. Signal transmission and reception are accomplished through the antennas by the vector network analyzer (ROHDE & SCHWARZ ZNB 40 10 MHz–40 GHz).

The anechoic boxes were fixed in place. The spacing between the anechoic boxes was 1 m, with a distance of 1.3 m between the two antennas. This separation was deemed sufficient to satisfy the far‐field criterion. Following each adjustment of the incidence angle, a background measurement was performed without the sample. This was done to establish a suitable time‐domain gate for each angle width. Finally, the sample measurements were normalized using the background measurement data. It is worth noting that the fiber‐reinforced plastic frame used to support the OMSM sample, along with the RF spillover effect, introduces unavoidable measurement errors at extreme incident angles. To account for this factor, the test specimen was made sufficiently large to ensure that energy within the frequency band is concentrated at the geometric center, with near‐zero attenuation at the edges and negligible spillover of the incident wave. Consequently, the test data for RF transmission at extreme angles of incidence is accurate.

Figure 6d,e show that the measured data now agree well with the simulation, confirming the successful achievement of the tri‐band transmission effect. Based on the measured data, in the low (2–3 GHz) and high (5–6 GHz) bands, the metasurface maintains $S_{21}$ above −1 dB for both TE‐ and TM‐polarized waves over an incident angle range of 0° to 60°, whereas in the middle band (3.8–4.1 GHz) the $S_{21}$ remains above −1.5 dB. For incident angles exceeding 60°, the $S_{21}$ slightly decreases at the band edges for TE‐polarized waves, and TM‐polarized waves retain excellent transmission efficiency. Regarding interference suppression, the two stopbands located between the three passbands exhibit an average of the $S_{21}$ below −10 dB under normal incidence. This stable out‐of‐band rejection performance is also evident in the simulation results shown in Figure 2b. Minor decreases in transmittance and frequency drifts in measurements can be attributed to the following: (i) Preparation process errors and variations in the inherent ohmic losses of the metal and (ii) incomplete elimination of incident wave spillover due to electromagnetic diffraction, particularly in lower frequency bands.

The optical properties of the OMSM were tested using a spectrophotometer (Lambda 1050, PerkinElmer). By adjusting the aperture of the photometer to precisely cover the structural region and averaging the transmittance values measured $\left(T_{\mathrm{mea}}\right)$ at different positions, results spanning the VIS (380–780 nm) and NIR (780–1800 nm) bands were obtained, as shown in Figure 6f. The average of $T_{\mathrm{mea}}$ is 94.24% at the VIS band and 94.44% at the NIR band. Transmittance measurements were also performed on unpatterned glass substrates. The equivalent transmittance $\left(T_{\mathrm{equ}}\right)$ of the OMSM was calculated using Equation (6), as shown in Figure 6f. $T_{c-g}$ is the transmittance of the unpatterned glass substrate, $A_{\mathrm{SMLU}}$ is the area of a single SMLU, and $A_{\mathrm{OMSM}}$ is the area of a single OMSM unit cell. The average of $T_{\mathrm{equ}}$ is 94.64% at the VIS band and 94.74% at the NIR band. The experimentally measured transmittance of the OMSM agrees excellently with the corresponding theoretical values, exhibiting a deviation of less than 0.5%. This small discrepancy mainly arises from the idealized assumptions in the equivalent model and the non‐ideal cross‐sectional profile and weak scattering losses of the fabricated metal lines. Notably, this deviation falls within the measurement repeatability error, further validating the accuracy of the experimental results and confirming the exceptionally high optical transparency of the OMSM. Furthermore, the OMSM exhibits excellent spectral uniformity (within ±1% across the aperture), maintains > 80% transmittance over 0°–60° (> 70% at 65°), and has an average haze of only 1.35%. See Supporting Information S1: Note 6 for details. Collectively, these results confirm the OMSM's outstanding optical quality for demanding imaging applications.

(6) $$T_{\mathrm{equ}}=T_{c-g}\times \frac{A_{\mathrm{SMLU}}}{A_{\mathrm{OMSM}}}$$

To further validate the practical optical detection capabilities of the OMSM, we conducted imaging experiments using a drone‐mounted system. As shown in Figure 7a, the OMSM was integrated into an optical imaging test system capable of imaging characterization across the 400–1700 nm spectral range. Experimental results demonstrate that its high optical transmittance is fully maintained under actual operating conditions on the drone platform, and no perceptible scattering or glare is introduced. The integrated system generates clear images with high detail resolution. The IC (calculated from mean grayscale values) and SNR (referenced to the windowless image in Figure 7b) were analyzed, revealing no significant degradation in the OMSM image (Figure 7d) compared to the TW image (Figure 7c), with differences of less than 0.3 dB in SNR and 1 in IC. Intensity extraction was also performed on the marked regions in Figure 7b–d. The normalized results are presented in Figure 7e–g, where the vertical axis (Position) spans from 0 to 508 pixels, corresponding to the bottom and top of the marked region, respectively. The intensity curve of the OMSM closely matches those of the windowless and TW images, revealing no significant differences. The results of the repeated tests also validate these analyses, as detailed in Supporting Information S1: Note 7. Moreover, modulation transfer function (MTF) measurements show a maximum difference of only 0.03 between the optical system with and without the OMSM, indicating a negligible impact (as detailed in Supporting Information S1: Note 6). These analyses confirm that even in complex optical paths, the metasurface pattern has a negligible impact on imaging quality. Collectively, these results demonstrate that the OMSM can operate stably on a dynamic platform under complex spectral environments, providing critical hardware support for achieving true cross‐spectral communication and optical detection integration in LAA.

**FIGURE 7:**
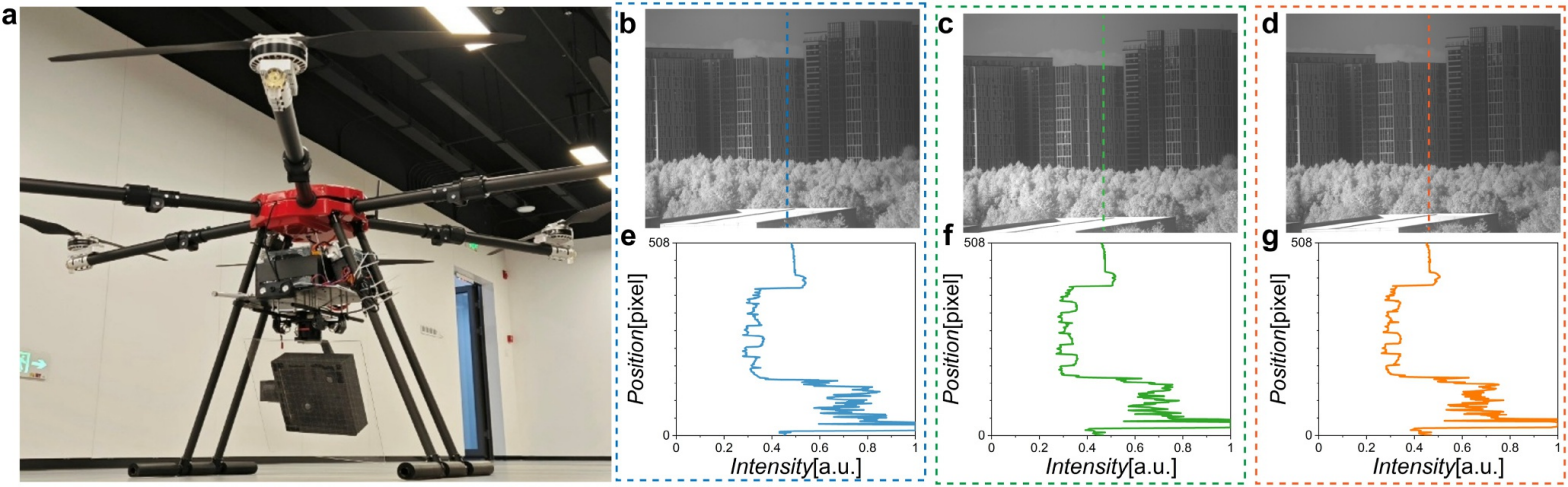
(a) The OMSM integrated into the LAA optical imaging system. The imaging results and intensity distribution at the marking position, when the optical imaging system (b, e) without the window assembly, (c, f) with the TW assembly, (d, g) with the OMSM assembly.

## 4 Conclusion

This study successfully designed, fabricated, and characterized an OMSM optical window device tailored for the applications of the LAA. To address the inherent physical tradeoff between RF selective transmission and broadband optical transparency, the core innovation lies in proposing a two‐dimensional spatial combination design strategy for the SMLU. This strategy synergistically optimizes the geometric configuration of subwavelength units to enable efficient transmission across target communication bands: 2–3 GHz, 3.8–4.1 GHz, and 5–6 GHz. Concurrently, ultra‐low‐duty‐cycle elongated metallic patterns ensure average transmittance exceeding 94% over the VIS and NIR spectra (380–1800 nm), achieving cross‐spectral functional compatibility at the physical layer. Compared with previous related reports, such as those summarized in Table 1, our results demonstrate outstanding performance in several aspects. In particular, in the optical domain, our sample exhibits superior optical transmittance, and in terms of imaging, the test conditions are closely aligned with actual operating conditions. The specific comparison metrics are as follows. In the RF domain, the comparison covers the passband range, the incident angle range over which stable transmission performance is achieved, and whether polarization insensitivity is achieved within this angular range. In the optical domain, the average transmittance in the VIS and NIR bands is compared. Additionally, the table compares the number of structural layers, the data source (simulation or experiment), and the types of operating conditions (laboratory or field).

**TABLE 1:** Comparison of RF and optical performance with prior works.

Reference	RF passband [GHz]	Incident angle [degree]	Polarization insensitivity	Average transmittance of VIS	Average transmittance of NIR	Number of layers	Data source	Operating condition
[34]	1–2	—	No	—	—	3	Sim.	—
	5–7							
	9.8–10.2							
	12.8–13.2							
[37]	1.9–1.94	0–80	Yes	—	—	2	Exp.	Laboratory
	2.06–2.12							
[41]	2.2–3.2	0–80	Yes	76.5%	—	3	Exp.	Laboratory
	4.4–5.2							
[42]	25–31	0–80	Yes	74%	67%	11	Exp.	Laboratory
[43]	25–35	0–80	Yes	80%	18%	7	Exp.	Laboratory
[50]	3 & 4.8	0–30	Yes	—	—	3	Exp.	Laboratory
[51]	2.5–3.8	0–60	Yes	—	—	4	Exp.	Laboratory
This work	2–3	0–70	Yes	94.2%	94.4%	2	Exp.	Laboratory& field
	3.8–4.1							
	5–6							

Experimental validation demonstrates that the OMSM sample (355× 355 ${\text{mm}}^{2}$) fabricated via large‐area low‐cost wet‐etching processes exhibits outstanding and robust performance. Its RF filtering characteristics exhibit favorable wide‐angle stability and polarization insensitivity within an incident angle range of 0°–60° for the passbands; beyond 60°, only slight attenuation occurs at the band edges. This agrees well with the simulation. Moreover, the optical imaging quality remains superior following integration with drone platforms. Our work provides a high‐performance, highly reliable, and easily manufacturable integrated solution for addressing the challenge of coordinating communication and optical sensing in the LAA. This advances the transition of compatible control metasurface from proof‐of‐concept to practical engineering applications, offering broad prospects for integration into next‐generation intelligent aviation platforms featuring integrated communication, sensing, and other advanced technologies.

## 5 Methods

*Fabrication of the OMSM Sample:* After an aluminum film with a thickness of 1 μm was deposited onto a clean bare glass substrate, a positive photoresist (AZ 1500) was spin‐coated at 2000 rev ${\text{min}}^{-1}$ for 30 s, yielding a layer approximately 0.52 μm thick. The photoresist was patterned by exposure through a photomask, followed by development to expose the underlying aluminum film in the designated regions. The exposed aluminum was then removed by wet etching in a phosphoric acid‐based etchant for 30 min. This step transferred the photoresist pattern into the aluminum layer, thereby defining the final metallic structures. Finally, the sample was immersed in acetone to strip the residual photoresist, followed by rinsing with deionized water and drying under a nitrogen flow.

*Full‐Wave Simulation:* Full‐wave simulation was performed using the frequency domain solver in CST Studio Suite 2022. A unit cell model of the OMSM was constructed. Subsequently, the boundaries in the *x* and *y* directions were set to “unit cell” to simulate an infinite periodic array. The boundary conditions in the *z*‐direction were set to “open (add space)”. The RF signal was incident as a plane wave.

*Optical Imaging Experiment:* The imaging test was conducted at 30${}^{\circ}31^{\prime }59^{\prime \prime }$ N, 104${}^{\circ}0^{\prime }0^{\prime \prime }$ E, with the system positioned approximately 19 m above ground level. Data acquisition commenced at 17:00. The imaging test was carried out over a spectral range of 400–1700 nm. The sensor featured a pixel pitch of 5.6 μm and an array size of 640 × 512 pixels, and the imaging lens had a focal length of 0.15 m with an f/3 aperture. The captured scenes comprised typical ground targets, including vegetation and buildings. The exposure settings were initially auto‐adjusted according to the ambient illuminance. To guarantee a fair comparison, these settings were then kept strictly identical across all control experiments—those performed without a window and those using the TW—throughout the entire measurement period.

## Author Contributions

**He Lin:** conceptualization, formal analysis, supervision, writing – review and editing, writing – original draft, visualization. **Yingchi Lin:** conceptualization, validation, formal analysis, writing – original draft, writing – review and editing, visualization. **Ling Wang:** investigation, validation. **Yuyao Wang:** data curation, investigation. **Ningrui Xiao:** data curation, investigation. **Da Mu:** supervision, resources. **Xiaoliang Ma:** conceptualization, methodology. **Cheng Huang:** resources, supervision. **Xiangang Luo:** methodology, funding acquisition, project administration.

## Funding

This work was supported by the National Key Research and Development Program of China (2023YFB4606105 and 2024YFA1210500). National Natural Science Foundation of China (NSFC) Fundamental Research Center Projects (52488301); Key Research Program of Frontier Sciences of the Chinese Academy of Sciences (ZDBS‐LY‐JSC030); and Western Light Program of the Chinese Academy of Sciences (xbzg‐zdsys‐202402).

## Conflicts of Interest

The authors declare no conflicts of interest.

## Supporting information


Supporting Information S1

## Data Availability

The datasets generated and/or analyzed during the current study are available from the corresponding author upon reasonable request.
