# Supplementary material for: Multispectral Shared‐Aperture Metasurface With RF Wide‐Angle and Polarization Stability for Tri‐Band Transmission and High‐Fidelity Imaging
Source: Nanophotonics. 2026 Jun 22;15(12):e70171. doi: 10.1002/nap2.70171 (PMC13642560; doi:10.1002/nap2.70171)
Supplement: Supplementary file 1 [file NAP2-15-e70171-s001.docx]

Supporting Information

Multispectral shared-aperture metasurface with RF wide-angle and polarization stability for tri-band transmission and high-fidelity imaging

He Lin^1,2,4^, Yingchi Lin^1,3^, Ling Wang^5^, Yuyao Wang^1,3^, Ningrui Xiao^1,4^, Da Mu^3^, Xiaoliang Ma^1,4*^, Cheng Huang^1,4*^, and Xiangang Luo^1,4*^

1 State Key Laboratory of Optical Field Manipulation Science and Technology, Institute of Optics and Electronics, Chinese Academy of Sciences, Chengdu, 610209, China.
2 School of Automation Engineering, University of Electronic Science and Technology of China, Chengdu 611731, China.
3 School of Optoelectronic Engineering, Changchun University of Science and Technology, Changchun, 130022, China.
4 College of Materials Science and Opto–Electronic Technology, University of Chinese Academy of Sciences, Beijing 100049, China.
5 The 722 Research Institute of China State Shipbuilding Co., Ltd., Wuhan, Hubei, 430205, China.

*Corresponding author: State Key Laboratory of Optical Field Manipulation Science and Technology, Institute of Optics and Electronics, Chinese Academy of Sciences, Chengdu, 610209, China,

Email Address: maxl@ioe.ac.cn, huangc@ioe.ac.cn, lxg@ioe.ac.cn

**Supplementary Note 1**

The *S*_21_ and *S*_11_ of the square ring, the T-shaped, and the circular ring structures at others frequency bands.

**Figure S1.** The *S*_21_ and *S*_11_ of the square ring at (a) middle, and (b) high frequency band. The *S*_21_ and *S*_11_ of the T-shaped structure at (c) low frequency band, and (d) high frequency band. The *S*_21_ and *S*_11_ of the circule ring at (e) low frequency band, and (f) middle frequency band.

**Supplementary Note 2**

By varying the parameter combinations, the entire operating frequency band can be adjusted, as illustrated in **Figure S2**a,b. As shown in Figure S2c–e, varying the linewidth *w* shifts the peak frequencies within the three transmission bands, but does not significantly improve the transmission efficiency at these peak frequencies. With regard to the period *P*, as presented in Figure S2f–h, its variation has a minor effect on the circular-ring substructure because it does not alter the current path but merely changes the periodic dimension of the structure. Consequently, the high frequency band is only weakly affected, whereas the low and middle frequency bands are influenced more significantly.

**Figure S2.** (a) Achieving a left-shift phenomenon across the entire operating frequency band through parameter combination changes, with corresponding structural parameters being *L*_1_ = 4.2 mm, *L*_2_ = 10.3 mm, *R* = 6.3 mm. (b) Achieving a right-shift phenomenon across the entire operating frequency band through parameter combination changes, with corresponding structural parameters being *L*_1_ = 4.7 mm, *L*_2_ = 10.3 mm, *R* = 7 mm. Effects of parameter variation on the value of *S*_11_ and *S*_21_ across different frequency bands: *w* in the (c) low, (d) middle, and (e) high frequency bands; *P* in the (f) low, (g) middle, and (h) high frequency bands.

**Supplementary Note 3**

As shown in **Figure S3**, the transmission phase difference between TE- and TM-polarized waves remains small and stable in three passbands (2-3, 3.8-4.1, and 5-6 GHz) over the entire range of incident angles (0-80°), with only a slight increase in the difference at the band edges at large incident angles.

**Figure S3.** The phase difference between TE- and TM-polarized waves varies with vary incident angle at (a) low, (b) middle, and (c)high frequency band.

**Supplementary Note 4**

The calculation methods for the impedance of different materials and the surface admittance of the metal pattern.

The impedance values of free space and compostie glass under different conditions are shown in Equation 1,2.

Following full-wave simulation of the OMSM, the transfer matrix *M_OMSM_* of the OMSM can be derived from the scattering matrix *S_OMSM_*. The expressions for the transfer matrix and scattering matrix are shown in Equation 3,4, with the specific conversion method detailed in Equation 5–8. After obtaining the transfer matrix *M_OMSM_*, the surface admittance *Y_m-p_* of the metal-pattern can be calculated using Equation 9.

**Supplementary Note 5**

A tolerance analysis was carried out based on full-wave simulations. For variations in metal linewidth *w*, conditions ranging from under-etching to over-etching were simulated, with the linewidth swept over a range of ±0.01 mm from the design value (covering the ±0.005 mm fabrication error). Meanwhile, substrate thickness fluctuations were considered by offsetting the glass substrate thickness *H* by ±0.01 mm from its design value. The simulation results, shown in **Figure S4**a,b, demonstrate that within the above-mentioned fabrication tolerance range, the *S*_21_ variations in the three passbands (2–3 GHz, 3.8–4.1 GHz, and 5–6 GHz) are all below 0.5 dB, and the out-of-band rejection remains below −10 dB. Regarding optical transmittance, the extremely low duty cycle of the pattern makes it almost insensitive to minor linewidth variations.

In addition, experimental tests on mechanical robustness and environmental durability were conducted. The OMSM samples were sequentially subjected to random vibration, equivalent aerodynamic load, damp heat, temperature cycling between −10 °C and +60 °C, and salt spray testing. After all tests, optical microscopy was performed on critical areas of the OMSM unit cell (connection areas between structural elements, T-shaped structures, and local arc segments of the ring structure). The results are shown in Figure S4d–f: the metal linewidth remains precisely within ±0.005 mm of the design value (i.e., within the tolerance range), and no evident damage such as cracking, warping, peeling, or corrosion is observed. This demonstrates that the low-duty-cycle pattern possesses excellent structural stability and environmental resilience.

**Figure S4.** (a) Effect of metal linewidth *w* and (b) substrate thickness *H* variations on RF performance. Microscopic images and measurement results of the structures after random vibration, equivalent aerodynamic load, damp heat, temperature cycling, and salt spray tests: (c) connection area of the unit cell, (d) T-shaped structure, (e) local area of the circular ring structure (arc-shaped line segment).

**Supplementary Note 6**

As shown in **Figure S5**a–d, a resolution target imaging test was carried out using a self-built optical system. A comparison of the results obtained with and without the OMSM window reveals that the maximum difference in MTF is only 0.03, indicating a negligible impact of the window on the system resolution. The detailed parameters of the MTF testing system are as follows: collimator focal length, 1.3 m; collimator aperture, 0.13 m; camera operating wavelength range, 400–1700 nm; lens focal length, 0.15 m; detector pixel size, 5.6 μm; and pixel array, 640 × 512. In addition, spectral transmittance at different positions within the clear aperture, spectral transmittance at different incident angles, and haze in the visible band were measured for the OMSM sample, with the results presented in Figure S5e–g. The uniformity of the spectral transmittance across the aperture is within ±1%, confirming excellent spectral uniformity over the entire aperture. Within the 0°–60° incident angle range, the visible–near-infrared transmittance remains above 80%, and it stays above 70% in the 60°–65° range, demonstrating favorable wide-angle light transmission performance. The average haze is only 1.35%, with a maximum of only 1.6%. Such low haze indicates that the extremely low-fill-factor metallic microstructure pattern introduces only minimal scattering, generates very little stray light and is highly compatible with downstream imaging systems. All the above spectral transmittance and haze measurements were performed using a spectrophotometer (Lambda 1050, PerkinElmer).

**Figure S5.** (a) Photograph of the optical system setup used for MTF measurement. Light is collimated by a collimator, passes through the OMSM, and then enters the imaging system. The resolution test target is placed at the focal plane of the collimator objective (near the light source side). (b) Target image acquired without the OMSM and (c) with the OMSM placed in front of the imaging system. (d) MTF values extracted from different positions of the target image and their corresponding spatial frequencies (defined as N, in lp/mm, where lp/mm denotes line pairs per millimeter). (e) Optical transmittance at different positions within the OMSM aperture. (f) Optical transmittance of the OMSM in the 380–1800 nm band as a function of incident angle. (g) Haze value of the OMSM in the 380–780 nm band.

**Supplementary Note 7**

Repeated imaging tests on the same target (**Figure S6**) demonstrate that OMSM imaging exhibits no significant degradation compared with TW imaging. In the first test (Figure S6a–c), the differences in SNR and contrast between OMSM and TW imaging are less than 0.6 dB and 1.1, respectively; in the second test (Figure S6g–i), the differences are less than 0.3 dB and 0.9, respectively. Intensity extraction was performed on the marked regions, and the resulting intensity profiles are presented in Figure S6d–f and j–l. The intensity curves of OMSM closely match those of the TW and windowless configurations, revealing no significant differences. These consistent results across repeated measurements further confirm the robustness of the OMSM, with no observable degradation.

**Figure S6.** The imaging results and intensity distributions at the marked positions under different configurations. (a, d) and (g, j) without the window assembly; (b, e) and (h, k) with the TW assembly; (c, f) and (i, l) with the OMSM assembly.
